# An Overview of Inter-Tissue and Inter-Kingdom Communication Mediated by Extracellular Vesicles in the Regulation of Mammalian Metabolism

**DOI:** 10.3390/ijms24032071

**Published:** 2023-01-20

**Authors:** Carlos Castaño, Anna Novials, Marcelina Párrizas

**Affiliations:** 1 Pathogenesis and Prevention of Diabetes Group, Instituto de Investigaciones Biomédicas August Pi i Sunyer (IDIBAPS), 08036 Barcelona, Spain; 2 Pathogenesis and Prevention of Diabetes Group, Centro de Investigación Biomédica en Red de Diabetes y Enfermedades Metabólicas (CIBERDEM), 08036 Barcelona, Spain

**Keywords:** diabetes, obesity, metabolism, inter-kingdom crosstalk, extracellular vesicles, gut microbiota, probiotics, microRNAs

## Abstract

Obesity and type 2 diabetes are associated with defects of insulin action in different tissues or alterations in β-cell secretory capacity that may be triggered by environmental challenges, inadequate lifestyle choices, or an underlying genetic predisposition. In addition, recent data shows that obesity may also be caused by perturbations of the gut microbiota, which then affect metabolic function and energy homeostasis in the host. Maintenance of metabolic homeostasis in complex organisms such as mammals requires organismal-level communication, including between the different organs and the gut microbiota. Extracellular vesicles (EVs) have been identified in all domains of life and have emerged as crucial players in inter-organ and inter-kingdom crosstalk. Interestingly, EVs found in edible vegetables or in milk have been shown to influence gut microbiota or tissue function in mammals. Moreover, there is a multidirectional crosstalk mediated by EVs derived from gut microbiota and body organs that has implications for host health. Untangling this complex signaling network may help implement novel therapies for the treatment of metabolic disease.

## 1. Introduction

The prevalence of non-communicable metabolic diseases is growing worldwide. Of these, obesity is the primary culprit, and it has been one of the major public health problems for decades, requiring novel nutritional or medical solutions [1]. Obesity rates are constantly on the rise, and it has been estimated that about 60% of the adult population will be overweight or obese by 2030 [1]. The global incidence of type 2 diabetes (T2D) is increasing in parallel [2], as it is closely related to obesity. Indeed, the term “diabesity” has been coined to refer to those cases when T2D is caused by obesity [3]. Moreover, obesity and T2D are associated with an increased risk of developing cardiovascular disease (CVD), mental disorders, cancer, and even infectious diseases such as COVID-19 [4].

To maintain metabolic homeostasis in complex organisms such as mammals, multiple organs and cell types must act in concert, and for that, they need to communicate with one another. When communication fails or is somehow disturbed, metabolic disease ensues. In the case of T2D, specific defects of insulin action or alterations in β-cell secretory capacity may underlie the development of the disease in different individuals. Indeed, extensive phenotyping of patients has consistently identified abdominal obesity, hepatic steatosis, or genetic alterations associated with deficient β-cell function as the key triggering defects [5,6,7]. Maladaptation of a tissue or cell type to environmental challenges may then influence other tissues through alterations of its secretory landscape, ultimately leading to impaired global metabolic homeostasis and masking the original malfunction [8]. Obesity induced by increased energy uptake and low physical activity is often associated with enlarged, hypertrophic white adipocytes that fail to adequately store the excess fat, leading to lipid spillover and ectopic accumulation in other tissues and decreasing insulin sensitivity [9]. Alterations of hepatic physiology caused by a diet enriched in processed food or high-fructose corn syrup can trigger hepatic steatosis that may progress to metabolic dysfunction-associated fatty liver disease (MAFLD), cirrhosis, or even hepatic carcinoma [10]. Exercise, alternatively, by increasing lean mass and basal metabolic rate, improves metabolic homeostasis [11]. In all these cases, the adipose tissue, the liver, or the muscle release humoral factors or extracellular vesicles (EVs) that may affect the function of neighboring and removed cells [8].

Importantly, in addition to lifestyle and genetic factors, obesity may also result from a perturbation of the gut microbiota, which then affects metabolic function and energy homeostasis in the host [12]. The gut microbiota is the set of microbes that colonize the gastrointestinal tract of mammals. In the case of humans, the gut microbiota is mainly composed of five different families: Bacteroidetes, Firmicutes, Actinobacteria, Proteobacteria, and Verrucomicrobia [13]. Remarkably, the gut microbiota establishes a mutualistic relationship with the host, participating in essential processes such as gut permeability, metabolism, and the physiology of the immune system [14]. Moreover, the gut microbiota can communicate with the central nervous system (SNC), participating in the regulation of hunger and appetite [15]. Environmental and endogenous factors such as dietary habits, aging, or antibiotic overuse may modify gut microbiota composition [16]. Alterations of gut microbiota homeostasis, referred to as dysbiosis, can promote overgrowth of pathogenic species, thereby playing a major role in the pathology of different diseases, including inflammatory bowel disease (IBD), CVD, neurological or autoimmune disorders, and colorectal cancer [17]. Recent studies have proposed that, whereas a healthy gut microbiota exerts a positive effect on health, dysbiosis may participate in the onset and progression of obesity and T2D [18,19]. In this sense, interventions on gut microbiota, including dietary interventions, bariatric surgery, or fecal transplantation, are postulated as novel, promising approaches to treating these metabolic disorders [20]. However, a better understanding of the mechanisms underlying the interaction between the gut microbiota and the host is necessary to identify the relevant targets.

**Figure 1:**
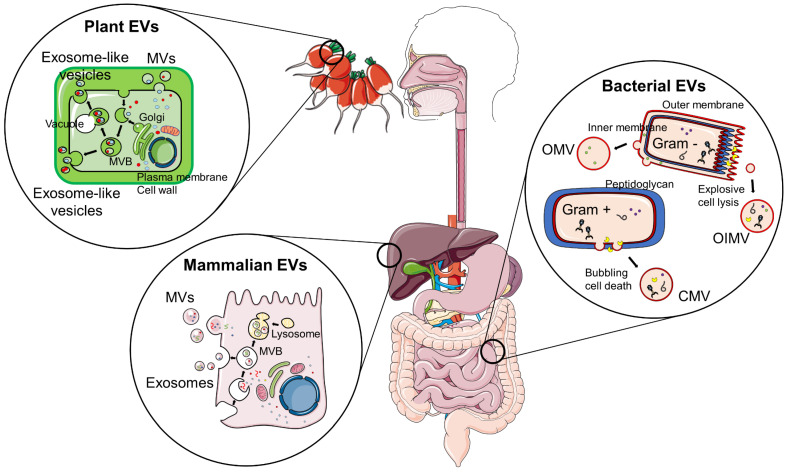
EV biogenesis in plants, mammals, and bacteria. Aside from apoptotic bodies generated upon programmed cell death, plant and mammalian cells release two main types of EVs: MVs resulting from plasma membrane evaginations and exosomes formed when the membrane of early endosomes invaginates in the intracellular space, engulfing a portion of the cytoplasm and giving rise to a multivesicular body (MVB) that may then fuse with lysosomes to recycle cellular components or with the plasma membrane, hence releasing the exosomes to the extracellular space. Alternatively, Gram-negative bacteria have two routes for bEV formation: blebbing of the outer membrane caused by cell cover disturbances leads to the production of OMVs that do not contain cytoplasmic components, whereas explosive cell lysis initiated by phage-derived endolysin that degrades the peptidoglycan cell wall results in cells bursting and releasing membrane fragments that self-assemble into OIMVs, which contain cytoplasmic components. In Gram-positive bacteria, endolysin triggers bubbling cell death, which is induced by DNA-damaging stress and results in CMVs that contain membrane and cytoplasmic components.

Recently, EVs have emerged as crucial players in inter-organ and inter-kingdom crosstalk, including the communication established between the gut microbiota and its mammalian hosts [21]. Remarkably, EVs found in edible fruits and vegetables or in milk have also been shown to influence the gut microbiota or tissue function in humans and mice [21]. EVs are released by most cell types and have been identified in all domains of life. In mammals, depending on their biogenesis and size, EVs have been classified into exosomes, microvesicles (MV), and apoptotic bodies [22]. Exosomes have an endocytic origin and range in size from 30 to 150 nm (Figure 1). By contrast, MV are formed from plasma membrane evaginations and have a diameter ranging from 100 to 1000 nm. Finally, apoptotic bodies originate from cellular apoptosis, may contain cellular DNA and organelles, and range from 50 to 5000 nm. Plant cells secrete EVs as well, including membrane-derived MVs and vesicles with a biogenesis, content, and morphology similar to that of mammalian exosomes, usually called exosome-like nanovesicles (Figure 1) [23]. EVs have also been identified in bacteria. Depending on their origin, bacterial EVs (bEVs) from Gram-negative bacteria, which possess a thin peptidoglycan layer sandwiched between a double lipid membrane, may be outer-membrane vesicles (OMVs) or outer-inner membrane vesicles (OIMVs). In the case of Gram-positive bacteria, which possess a lipid membrane and an external peptidoglycan wall, bEVs are cytoplasmic-membrane vesicles (CMVs) [24] (Figure 1). Eukaryotic EVs and bEVs differ in factors such as biogenesis, composition, and tolerance to high temperatures. However, bEVs have many similarities with eukaryotic EVs in size, morphology, and stability. Importantly, a common characteristic among all EVs is the ability to carry and transfer bioactive molecules among cells. EV cargo is composed of a variety of bioactive molecules, including lipids, proteins, RNA, and DNA [22]. Among those, microRNAs (miRNAs) have raised special interest, as they can modulate gene expression in unrelated organisms, including plants–mammals, plants–bacteria, and mammals–bacteria [25]. Remarkably, miRNA-like molecules identified in bacteria have a similar size to eukaryotic miRNAs and can also be encapsulated and secreted in bEVs to protect them from degradation.

In this review, we will discuss the multidirectional crosstalk mediated by EVs derived from gut microbiota and the body organs in the context of obesity and related metabolic diseases. Moreover, we will examine the effects of EVs derived from food and probiotics/prebiotics on gut microbiota and the potential implications of this interaction on host health. Untangling this complex signaling network may help implement novel therapies for the treatment of metabolic diseases.

## 2. Inter-Organ Communication Mediated by EVs

### 2.1. EVs from the Adipose Tissues

#### 2.1.1. White Adipocytes and Macrophages

Enlarged white adipose tissue (WAT) mass in obesity is often associated with tissue dysfunction manifested as an anomalous secretion pattern. Increased release of pro-inflammatory mediators and decreased secretion of adiponectin, for instance, are hallmarks of obesity [26]. However, the adipocytes and other cell types conforming to the WAT also release EVs that can be detected circulating in the blood. Although well-characterized tissue-specific EV markers are still lacking, vesicles enriched in proteins highly expressed in the adipose tissues, such as the transporter fatty acid binding protein (FABP) 4 or the hormone adiponectin, have been identified in human blood [27]. Moreover, adipose tissue, including both white and brown varieties, has been described as the main source of exosomal miRNAs circulating in the blood [28]. The strategy used to reach this conclusion, by deleting the miRNA processing enzyme DICER1 from mature adipocytes, did not modify the number of circulating blood exosomes, although it dramatically decreased the miRNAs associated with them. Interestingly, other authors overexpressed tdTomato fluorescent protein specifically in adipocytes, resulting in the release of fluorescent exosomes. The low ratio of tdTomato to the exosomal membrane protein CD63 in blood exosomes suggested that adipose exosomes represent a small fraction of those detected in blood [29]. These observations would be in accordance with another study concluding that the ratio of miRNAs to exosome numbers in blood is so low that probably only a small fraction of exosomes carry miRNAs [30]. Hence, adipose exosomes would be particularly enriched in miRNAs, even though they represent a low percentage of the vesicles circulating in the blood. Analysis of the RNA content of circulating EVs and correlation with RNA profiles from different tissues also suggested that the vast majority of EVs in blood come from hematopoietic cells [31], although EVs from peripheral tissues such as the liver could also be detected. Importantly, the hepatic EV fraction could still differentiate healthy patients from those with liver disease [31]. Similarly, many reports describe increased release of exosomes to the circulation by the obese adipose tissue [29]. Interestingly, knocking down adipocyte histone deacetylase SIRT1 results in obesity and increased exosome release by the adipocytes due to reduced autophagy, with the secreted exosomes then participating in setting off insulin resistance in both the adipose tissue and the liver [32].

Recently, extensive characterization of the protein and miRNA content of EVs derived from different cell lines and primary cells representing white and brown adipocytes, hepatocytes, skeletal muscle, and endothelial cells identified different molecular signatures for each cell type. Interestingly, tetraspanins CD9, CD63, and CD81, considered classical markers of EVs and of exosomes in particular and frequently used to assist in their isolation or detection, are not equally enriched in EVs from all cell types. CD63, for instance, is abundant in muscle EVs but is present only at low levels in hepatic, adipose, and endothelial EVs [33,34]. Importantly, the authors identified a set of proteins found in the EVs of all cell types examined, such as the enzymes glucose−6-phosphate isomerase (GPI) and enolase (ENO) 1 and the membrane protein colony-stimulating factor 1 receptor (CSF1R), which may prove to be of use in isolating and characterizing circulating EVs relevant to metabolic disease [33].

**Figure 2:**
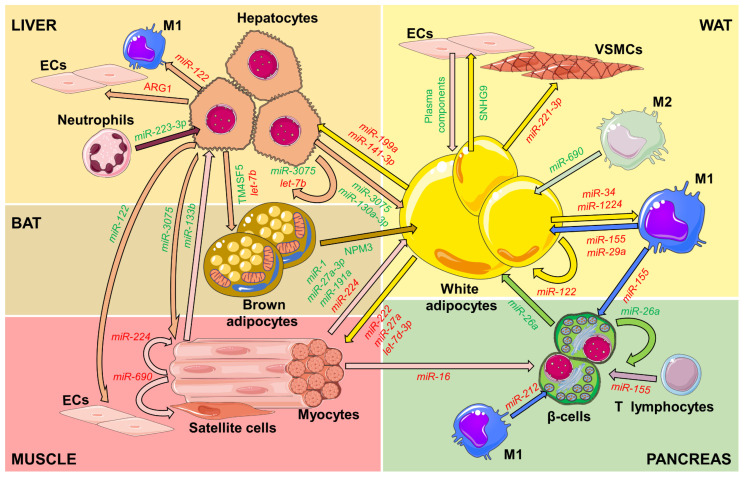
EVs and their cargo participate in inter-organ crosstalk to regulate metabolic homeostasis. EVs released by many cell types can be captured by acceptor cells in an autocrine and paracrine manner, initiating biological responses. In addition, EVs can go into the circulation and reach other organs, driving inter-organ crosstalk. M1: M1 macrophages; M2: M2 macrophages. See text for details.

Many reports have focused on studying the characteristics and effects of EVs released by the different cell types that constitute the WAT, particularly adipocytes and macrophages [35,36,37,38], but also other cell types (Table 1). In this regard, it has been shown that the endothelial cells (ECs) release vesicles transporting plasma components to the adipocytes [39]. In return, ECs receive adipocyte-released EVs containing long non-coding RNA small nucleolar RNA host gene (SNHG) 9. In ECs, SNHG9 interacts with and silences TNF receptor type 1-associated death domain protein (TRADD) mRNA, reducing inflammation and apoptosis (Figure 2) [40]. In addition, adipocyte-secreted adiponectin accumulates in ECs and enhances EV release by these cells, thus decreasing intracellular ceramides and improving EC function [41]. Adipocyte-released EVs also transfer *miR-221-3p* to vascular smooth muscle cells (VSMCs) to induce remodeling during obesity [42] and can also influence neighboring adipocytes in a paracrine manner. Hence, transmission of *miR-122* from mature adipocytes promotes adipogenesis of neighboring precursor cells by increasing the expression of sterol regulatory element binding transcription factor (SREBF) 1 [43], whereas vesicles from hypoxic adipocytes of obese subjects decrease glucose uptake of neighboring cells [44].

EVs also participate in crosstalk between adipocytes and macrophages, with vesicles released by obese adipocytes transporting neutral lipids to macrophages [29], blocking M2 polarization by transferring miRNAs such as *miR-34* or *miR-1224* [46,47], or inducing M1 polarization and foam cell formation [48]. In vitro, THP1 monocytes treated with anti-inflammatory interleukin (IL) 4 release exosomes that induce expression of *miR-21* and other miRNAs in 3T3-L1 adipocytes, resulting in enhanced insulin-stimulated glucose uptake, whereas lipopolysaccharide (LPS)-activated THP1 cells increase pro-inflammatory gene expression in adipocytes [82,83]. In vivo, M2 polarized anti-inflammatory macrophages release exosomal miRNAs such as *miR-690*, which have insulin sensitizing properties, whereas pro-inflammatory M1 macrophages release *miR-155* or *miR-29a*, which decrease insulin sensitivity [35,36,63]. Similar intra-organ communication between resident macrophages and tissue cells has been observed in other locations, such as the pancreatic islets, where M1 macrophages release exosomes enriched in *miR-212* that target histone deacetylase SIRT2 and decrease insulin secretion in acceptor β-cells [64]. However, exosomes from WAT macrophages can also affect the pancreatic β-cells by transferring *miR-155* and impairing insulin secretion while enhancing β-cell proliferation [65]. Similarly, exosomes from adipose tissue explants of healthy subjects or 3T3-L1 adipocytes induce proliferation of INS1 β-cells, whereas those from obese subjects induce cell death [84]. Moreover, serum exosomes from obese nondiabetic subjects carry low levels of omentin and inhibit β-cell proliferation without inducing apoptosis [85]. Direct communication between the WAT and the pancreas may then participate in the β-cell expansion and increased insulin secretion observed at the beginning of metabolic disease and the ultimate β-cell failure characteristic of advanced states of obesity and T2D.

In addition to pancreatic β-cells, adipocyte EVs can also affect other cells outside of the adipose depots, such as the muscle [38,49,52] or the liver [38,53,54,86,87]. Most reports show how EVs from aging or obese adipose tissue induce inflammation, insulin resistance, and toxicity in acceptor cells, contributing to disease spread. Transfer of exosomal *miR-222* or *miR-27a* from obese adipocytes to muscle, for instance, reduces insulin sensitivity [38,49], whereas *let-7d-3p*, enriched in EVs from aged adipocytes, induces sarcopenia by decreasing muscular stem cell proliferation [52]. In addition, increased *miR-199a* and decreased *miR-141-3p* in vesicles from adipose tissue of obese mice fed a high-fat diet (HFD) induce hepatic steatosis and insulin resistance [53,54].

Adipose EVs can also affect heart function. Indeed, EVs from stressed adipocytes have been shown to carry mitochondria that increase antioxidant pathways in accepting cardiomyocytes [50]. Interestingly, most communication events take place locally. Thus, EVs from epicardial adipocytes have a protective effect after myocardial infarction by attenuating cardiac remodeling through transference of adipsin to cardiomyocytes [51], whereas EVs from epicardial adipocytes of atherosclerotic heart disease patients carry *miR-3604* that blocks neuronatin expression in acceptor epicardial adipose stem cells to reduce adipogenesis [45]. Along similar lines, intramyocardial injection of vesicles isolated from the serum of diabetic patients exacerbates myocardial ischemia/reperfusion injury in the nondiabetic heart by transferring *miR-130b-3p* [88].

Remarkably, adipocyte-derived EVs may also mediate communication between WAT and the brain, promoting cognitive impairment by transferring *miR-9-3p* [55] or regulating hunger by communicating with hypothalamic neurons [89]. Furthermore, in obese mothers, adipocyte exosomes may affect fetal health [90]. Injection of EVs from the visceral WAT of obese mice into pregnant female mice resulted in increased inflammation in the placenta and fetal heart dysfunction [90].

Finally, exosomes from adipocytes also provide a link between obesity and cancer as they can modulate the function of tumor cells in breast cancer, prostate cancer, melanoma, or lung cancer, for instance [91,92,93,94,95]. By activating hypoxia-inducible factor (HIF) 1α, decreasing cyclin-dependent kinase inhibitor (CDKN) 2A activity, or transferring matrix metalloproteinase (MMP) 3 or fatty acid oxidation enzymes and substrates to tumor cells, EVs from obese adipose tissue enhance their malignancy, proliferation, and invasiveness.

#### 2.1.2. Brown Adipocytes

Brown adipocytes differ from white adipocytes in their origin from common precursors to muscle cells [96,97]. However, brown-like adipocytes can also arise in WAT by either trans-differentiation of mature white adipocytes or brown differentiation of white adipocyte precursors, in a process known as beigeing or browning [98]. In contrast to white adipocytes, brown adipocytes contain multilocular lipid droplets and are rich in mitochondria, which express uncoupling protein (UCP) 1, thus contributing to energy expenditure and thermogenesis. By this ability and through their secretory capacity, brown adipose tissue (BAT) activation has been considered a potential strategy for the treatment of obesity [99,100]. Accordingly, EVs from BAT have been described as exerting beneficial effects. EVs released by brown adipocytes carry proteins such as nucleophosmin (NPM) 3, long non-coding RNAs, or mitochondrial components that contribute to WAT browning in vitro or in vivo (Figure 2) [56,101,102]. BAT exosomes also ameliorate diabetic kidney disease by transferring *miR-30b* and thus blocking pro-fibrotic transcription regulators runt-related transcription factor (RUNX) 1 and snail family zinc finger (SNAIL) 1 [57]. Interestingly, BAT ablation decreases the beneficial effects of exercise. Knockdown of the small GTPase RAB27A in intrascapular BAT, which decreased EV release by the tissue, attenuated the exercise-induced cardioprotective effects [103], thus highlighting the need for tissue crosstalk to respond to environmental challenges.

#### 2.1.3. Mesenchymal Stem Cells

Mesenchymal stem cells (MSCs) are adult stem cells with significant anti-inflammatory and regenerative properties that have been used as cellular therapy for many ailments, including obesity and diabetes. Interestingly, EVs released by MSCs seem to reproduce some of the effects of MSCs but with a lower risk of tumorigenesis and offer the opportunity for cell-free therapy [22]. WAT is a frequently used source of MSCs due to its accessibility, and the effects of adipose MSC EVs on metabolic regulation have also been thoroughly examined. Adipose MSC EVs induce M2 macrophage polarization by transferring signal transducer and activator of transcription (STAT) 3 that then transactivates arginase (ARG) 1 [58], protect from osteoporosis by carrying osteoprotegerin (OPG), which blocks osteoclast differentiation [59], and decrease liver fibrosis by suppressing stellate cell activation [104]. Furthermore, MSC eVs protect from hepatic steatosis through the transference of *miR-627* [105] or *miR-223-3p* [60]. EVs from MSCs also induce beneficial effects upon diabetic complications, including nephropathy, by protecting podocytes from apoptosis [106] and inflammation [107]. In addition, they can accelerate diabetic wound healing by transferring *miR-21-3p* or *miR-126,* both of which activate the phosphatidyl-inositol 3 kinase (PI3K) signaling pathway [61,62]. Similarly, MSCs derived from the human umbilical cord ameliorate experimental non-alcoholic steatohepatitis (NASH) in mice by targeting the nuclear factor erythroid 2-related factor (NRF) 2 pathway [108]. Moreover, human umbilical cord MSCs EVs increase glucose uptake by adipocytes in vitro [109] and reduce blood glucose in experimental diabetic rats by increasing insulin signaling in muscle, glycogen accumulation in the liver, and decreasing β-cell apoptosis [110].

Interestingly, by comparing the proteome of EVs released by MSCs obtained from either bone marrow or adipose tissue, it was observed that molecules present exclusively in EVs from adipose MSCs were highly correlated to angiogenesis, whereas those expressed in EVs from bone marrow were preferentially involved in cellular proliferation [111]. These data suggest that the cellular source of the EVs is of high importance in determining the ultimate effects of the therapy. Further stressing this notion, EVs of adipose MSCs from subjects with type 1 diabetes (T1D) are enlarged, reduced in number, and have a higher percentage of CD9 [112], and EVs from bone marrow MSCs obtained from aged mice contain high levels of *miR-29-3p* as compared with those isolated from younger mice and induce insulin resistance in acceptor young mice [113].

### 2.2. EVs from the Muscle

Like the adipose tissues, the skeletal muscle is a source of humoral factors, in this case called myokines, that get distributed throughout the body and participate in cell communication, helping maintain metabolic homeostasis and adapting to the enhanced metabolic demands of exercise [114,115]. The muscle also releases EVs [114], and the number and cargo of these vesicles change during and after exercise or in the context of obesity [37,116,117]. In vitro, mechanical strain enhances EV release by C2C12 cells [118]. Exercise-associated vesicles often accumulate in the liver [66,117], providing a means for coordinating tissue activity in a context of high energy requirements. We recently showed that implementation of high-intensity interval training (HIIT) in mice results in the release of exosomal *miR-133b* and other muscle-enriched miRNAs that then contribute to the improvement of metabolic homeostasis and insulin sensitivity induced by exercise by decreasing the expression of insulin-regulated transcription factor forkhead box (FOX) O1 in the liver (Figure 2) [66]. Similarly, other reports described the effects of muscle-derived EVs released during exercise in promoting WAT lipolysis by transferring *miR-1* [67] or browning by decreasing *miR-191a* in EVs [68]. In HFD mice, exercise decreases *miR-27a-3p* in EVs from skeletal muscle, also contributing to WAT browning [119]. In contrast, EVs derived from atrophic muscle fibers of aged mice carry *miR-690* that inhibits satellite cell differentiation [69], and atrophic insulin-resistant muscle from obese mice releases fewer EVs, which are enriched in proteins involved in lipid oxidation and miRNAs with nuclear location signals, such as *miR-224*, that target genes encoding proteins with nuclear activities in recipient adipocytes and neighboring muscle cells [37]. The quadriceps muscle of obese mice fed a palmitate-rich diet releases *miR-16* in EVs that are captured by pancreatic β-cells, increasing proliferation [70] EVs released by the muscle have also been shown to communicate with the bone [120,121] or ECs [122].

### 2.3. EVs from the Liver

The liver is also a source of EVs that participate in metabolic regulation. Indeed, hepatic EVs carry *miR-130a-3p* that can be captured by adipocytes in vitro, where it targets the PH domain and leucine-rich repeat protein phosphatase (PHLPP) 2 [123], whose silencing has been shown to increase insulin sensitivity by favoring peroxisome proliferator-activated receptor (PPAR) α activity in adipocytes (Figure 2) [71]. Furthermore, injection of exosomes carrying *miR-130a-3p* into *miR-130*^−/−^ mice correlates with increased insulin sensitivity [123]. Liver EVs also enhance glucose uptake by brown adipocytes by transferring the transmembrane protein TM4SF5 and improving insulin sensitivity [72]. Even hepatocyte-derived exosomes from early-onset obese mice have a protective effect and promote insulin sensitivity by transferring *miR-3075* to acceptor cells in the muscle, the adipose tissue, or the liver itself [73].

However, in frank obesity or when liver disease is present, hepatic EVs contribute to further disturbing metabolism. Hence, hepatocytes treated with palmitic acid release EVs enriched in *let−7b*, whose overexpression enhances fatty acid transport by hepatocytes while reducing brown adipocyte thermogenesis. Interestingly, release of *let−7b* was regulated by the transforming growth factor (TGF) β pathway in hepatocytes [77], a key player in the development of liver disease [124], and it was absent in mice with hepatic-specific deletion of the receptor TGFBR2. These liver-specific knockout mice were also resistant to steatosis and obesity [77]. Hepatocyte EVs also carry ARG1 and contribute to the increase of arginase activity in the plasma of HFD mice [125,126], which is considered to initiate atherosclerosis.

One of the most characteristic hepatic-enriched miRNAs, *miR-122*, has been repeatedly shown to be increased in obese human and mouse plasma vesicles [76,127] and we have shown that it participates in the setting of the first steps of metabolic dysfunction associated with diabetes by targeting PPARα in WAT [76]. Similarly, *miR-122* released by lipid-exposed hepatocytes is captured by macrophages, inducing expression of pro-inflammatory genes in a process requiring MMP2 [128]. Hepatic exosomal *miR-122* also impairs cardiomyocyte function. Human plasma exosomes transport *miR-122* into mouse primary cardiomyocytes and impair mitochondrial ATP production and oxygen consumption. In obese mice, increased hepatic and circulating exosomal *miR-122* also inhibited cardiac mitochondrial function [74]. Alternatively, a recent report showed that exercise induces the release of exosomal *miR-122* by the liver, which is required for the ECs of the skeletal muscle to initiate angiogenesis. EV *miR-122* enhanced EC fatty acid utilization by targeting 1-acyl-sn-glycerol−3-phosphate acyltransferase (AGPAT) 1, hence favoring angiogenesis [75]. These data indicate that the effects of a miRNA often depend on context and highlight the need for limiting delivery specifically to target cells when using a miRNA mimic or inhibitor as therapy to avoid unwanted secondary effects.

Hepatic nonparenchymal cells also communicate by releasing EVs. Neutrophil EVs are enriched in anti-inflammatory *miR-223-3p,* which is transferred to hepatocytes and limits the progression of NASH. Interestingly, the presence of apolipoprotein (APO) E on neutrophil-derived EVs facilitated direct delivery to the hepatocytes by binding to the low-density lipoprotein receptor (LDLR) [78]. Conversely, EVs from the hepatic stellate cells carry the glucose transporter GLUT1 and the glycolytic enzyme pyruvate kinase (PK) M2 that induce a metabolic switch in neighboring quiescent stellate cells, resulting in the advancement of fibrosis [129].

### 2.4. EVs from Pancreatic β-Cells

The pancreatic islets are crucial regulators of metabolic homeostasis through the controlled release of insulin by β-cells or glucagon by α-cells, among other hormones and bioactive peptides. However, islet cells can also exert autocrine and endocrine effects through the release of EVs [130]. Hence, EV *miR-26a* released by β-cells targets regulators of insulin release and cell proliferation in the β-cell itself but also modulates peripheral insulin sensitivity (Figure 2) [79]. Exosomes from the β-cell line MIN6 injected into streptozotocin-induced diabetic mice improve the survival and glucose tolerance of the treated mice, as well as pancreas architecture and insulin content [131]. Pancreatic EVs from healthy subjects reduce amyloid formation in the pancreas, a hallmark of diabetes [132], but conversely, EVs containing islet amyloid polypeptide (IAPP) can reach hippocampal cells, inducing a pro-fission status of the mitochondrial network and providing a link between diabetes and neurodegenerative disease [80]

Rodent and human T lymphocytes release EVs with pro-inflammatory *miR-155* among other miRNAs that induce β-cell apoptosis and may participate in the development of T1D [81]. Alternatively, cytokine treatment of β-cell lines induces *miR-21* release in EVs, and serum *miR-21* is increased in NOD1 mice and T1D children as the disease progresses [133]. Furthermore, EVs from β-cells treated with cytokines promote a pro-inflammatory islet transcriptome in neighboring cells and may contribute to β-cell failure in diabetes [134].

Finally, tumor cells hijack organismal glucose and lipid metabolism to thrive; thus, it is not surprising that they can affect β-cell function by releasing exosomes carrying *miR-122* that targets PKM2 and decreases glycolysis, hence decreasing ATP-stimulated insulin release and impairing glucose metabolism to favor tumor growth [135].

## 3. Inter-Kingdom Communication Mediated by EVs

### 3.1. Environmental Factors Shaping Gut Microbiota and bEVs

**Figure 3:**
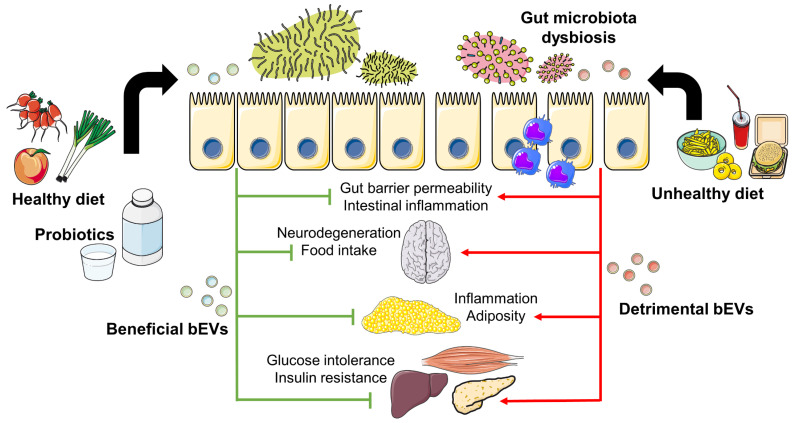
Impact of dietary patterns on gut microbiota composition and bEV profile and its effects on metabolic diseases. Unhealthy dietary patterns contribute to obesity and T2D by promoting gut microbiota dysbiosis. The increase in detrimental bEVs may then affect gut barrier permeability and intestinal inflammation. After gaining access to the systemic circulation, bEVs may disturb peripheral organ function, thus impairing metabolic homeostasis and promoting inflammation, thereby increasing the severity of metabolic disorders. Conversely, an increase of beneficial bEVs through a plant-based diet or probiotic supplements could be a strategy to treat metabolic disorders by counteracting dysbiosis, gut permeability, inflammation, metabolic homeostasis disturbances, and nervous system imbalance. See text for details.

The gut microbiota participates in the digestion of carbohydrates, lipids, and essential amino acids and plays a role in metabolism through the production of short-chain fatty acids (SCFAs) by colonic fermentation of dietary fiber and proteins. The beneficial effects of SCFAs on body weight, the regulation of satiety, glucose and lipid homeostasis, insulin sensitivity, and the maintenance of intestinal barrier strength have been well documented [136]. Moreover, other metabolites produced by the gut microbiota also reach the circulation of the host and have been shown to regulate gene expression in distant tissues, such as tryptophan-derived metabolites that control the expression of the *miR-181* family in WAT, in this way regulating energy expenditure and insulin sensitivity in mice [137]. Additionally, gut microbiota increase energy production from food, provide low-grade inflammation, and impact adipose tissue composition, hence playing a critical role in the development of obesity. Multiple factors such as diet, physical activity, medication, or bariatric surgery may affect the composition of the gut microbiota. Factors decreasing diversity lead to dysbiosis and, consequently, weight gain and obesity. Conversely, increased diversity favors a healthy metabolism (Figure 3).

Diet exerts a powerful influence on the composition, abundance, diversity, and metabolism of gut microbiota [138]. Promotion of a specific gut microbiota composition profile has been established for a wide range of diets [139]. In mice, HFD and high-sucrose (HSD) diets have different effects on gut microbiota. Some favorable bacteria are reduced in HFD, whereas more obesity-related bacteria are present after HSD feeding, thus suggesting that dietary sucrose impacts obesity to a greater extent than dietary fat [140]. In humans, vegetarian and Mediterranean diets prevent obesity by increasing diversity, particularly the abundance of beneficial bacteria, while decreasing inflammation-related bacteria [141,142]. High-quality whole grain consumption also reduces weight gain [143]. In fact, African children, who consume a high-fiber/low-fat diet, have higher microbial diversity and fewer pathogenic bacteria, with larger amounts of Bacteroidetes than European children, who have higher amounts of Firmicutes. Actually, obese subjects have a higher Firmicutes/Bacteroidetes ratio than individuals of normal weight, and this ratio correlates negatively with energy expenditure. Interestingly, there is a negative correlation between circulating *miR-122*, which, as mentioned, is usually increased in obese and diabetic patients, and the presence of *Bacteriodes uniformis* [144]. In contrast, a low-fiber/high-fat diet reduces microbial diversity and enhances gut inflammation, thus evidencing that the westernization of dietary habits induces microbial dysbiosis [145,146]. Therefore, gut microbiota shaping through dietary interventions could be an attractive, effective, and non-invasive strategy to prevent or treat obesity and diabetes.

Alternatively, dietary supplements that can prevent weight gain act at least in part by improving lipid and glucose metabolism through modifying gut microbiota composition. Both the antioxidant derivative of carotenoids astaxanthin and the medicinal mushroom *Antrodia cinnamomea* optimize the Firmicutes/Bacteroidetes ratio and increase *Akkermansia muciniphila* in obese mice [147]. Interestingly, direct transfer of *B. thetaiotaomicron* to mice fed a normal diet results in a reduction in total fat and also prevents weight gain in obese mice. Furthermore, the *Akkermansia* phylum counteracts inflammation and improves insulin sensitivity. The remarkable protective effect of *A. muciniphila* on metabolism is also reported in human studies, where it has been shown that it is able to reduce intestinal permeability, reinforce the immune system, and improve glucose homeostasis [148].

Remarkably, aerobic exercise also enhances bacterial diversity, mainly through SCFA producers. Obese adults following aerobic moderate-to-intense physical activity decrease the Firmicutes/Bacteroidetes ratio [149]. Furthermore, both metformin and bariatric surgery increase *Bacteroides* species [150,151,152,153]. Conversely, the overuse of antibiotics induces dysbiosis and correlates with an increased risk of developing inflammatory disorders and obesity [154].

Human gut microbiota continually produces bEVs that can enter the systemic circulation and elicit a variety of responses in the host, thus evidencing the role of bEVs in microbe-host cross-kingdom communication [155]. In this sense, bEVs may participate in immune system regulation, modulation of cerebral function, or the metabolism of peripheral tissues (Figure 3). Importantly, diet and other environmental factors influence the bEV profile and its effects on host physiology, thus contributing to the development or alleviation of metabolic disorders. For instance, intestinal succinate produced after administration of a high-protein diet (HPD) induces reactive oxygen species (ROS) production by the gut microbiota that enhances the release of bEVs in mice [156]. HFD mice also have an altered content of bEVs in their stool, showing a reduction in size and changes in the global protein content, marked by an increment in LPS due to the increase in bEVs derived from LPS-expressing *Proteobacteria* [157]. This higher percentage of LPS-containing bEVs could potentially be an important underlying mechanism orchestrating the metabolic disorders induced by HFD [158].

### 3.2. bEVs and Their Impact on Inflammation and Metabolism

Importantly, bEVs from beneficial species have a protective role against inflammatory responses (Table 2). *A. muciniphila* bEVs, in particular, dampen the secretion of pro-inflammatory cytokines induced by pathogenic *Escherichia coli* bEVs on a colon epithelial cell line, thus suggesting a potential role for *A. muciniphila* bEVs in regulating intestinal barrier permeability [159]. Similarly, *Odoribacter splanchnicus 57* bEVs possess immunomodulatory properties, mitigating the production of pro-inflammatory cytokines in enterocytes [160]. *E. coli Nissle (EcN) 1917* and *ECOR63* bEVs also protect the barrier function in human intestinal epithelial cell cultures [161]. The role of bEVs in modulating intestinal inflammation and gut barrier integrity has been further confirmed by several in vivo studies. For instance, oral administration of *A. muciniphila, EcN*, or *B. fragilis* bEVs attenuates the severity of colitis in mice [162,163,164]. Interestingly, bEVs not only exert immunomodulatory functions through their interaction with gut barrier cells but also communicate with intestinal immune system cells, thus influencing host immunity. In this regard, *Pediococcus pentosaceus* bEVs trigger immuno-suppressive responses in bone marrow-derived macrophages [165]. It should be highlighted that the induction of a moderate inflammatory bowel response is critical for gut barrier protection. In fact, bEVs with mild pro-inflammatory properties could be beneficial and contribute to gut barrier integrity and intestinal homeostasis. In this sense, *E. coli C25* bEVs promote interleukin secretion by intestinal epithelial cells [166], and *EcN* and *ECOR12* bEVs enhance the secretion of pro-inflammatory cytokines in human epithelial colorectal adenocarcinoma (Caco−2) cells, thus suggesting a role for LPS-carrying bEVs in the modulation of intestinal immune responses [167]. However, in the context of dysbiosis, increased pro-inflammatory bEVs may exacerbate inflammatory responses. Hence, *Faecalibacterium prausnitzii* bEVs cause gut barrier permeability and alter epithelial cells’ metabolic functions through the regulation of tight junctions and the expression of PPAR genes, as observed in Caco−2 cells [168]. This stronger pro-inflammatory response may ultimately harm the host, as in the case of colitis development in genetically susceptible mice that receive *B. thetaiotaomicron* bEVs [169].

Notably, bEVs also help connect intestinal immune responses and gut barrier integrity with host metabolic functions. Oral administration of *A. muciniphila* bEVs to HFD mice increases gut barrier integrity and ameliorates intestinal and adipose tissue inflammation, but also reduces food intake, body weight, and adiposity and improves glucose tolerance [170,171,172]. These data indicate that *A. muciniphila* bEVs could be a therapeutic tool to treat metabolic diseases such as obesity and diabetes [183]. Alternatively, changes in bEV profile and cargo occurring in dysbiosis can contribute to the onset and progression of metabolic diseases [184]. Remarkably, the alteration of the bEV profile is more severe than changes in gut microbiota composition. In a context of obesity-induced gut barrier permeability, bEVs and other microbiota-derived products can enter host circulation and reach insulin-sensitive tissues, evidencing the potential of bEVs as biomarkers for intestinal permeability [185]. Actually, accumulation of microbial DNA carried by bEVs in diverse organs of the host may lead to disease, such as adrenomedullary abnormalities and hypertension [177], liver steatosis and fibrosis [178], and development of obesity-associated tissue inflammation and insulin resistance in mice [179]. Accumulation of DNA-containing bEVs also promotes islet inflammation and β-cell abnormalities in obese mice [180]. *Pseudomonas panacis* LPS-containing bEVs, which are highly increased in the stool of HFD mice, blunt glucose metabolism after administration to mice by promoting insulin resistance in both skeletal muscle and adipose tissue [157]. Moreover, *Porphyromonas gingivalis* bEVs decrease hepatic glycogen synthesis in response to insulin, thus eliciting changes in glucose metabolism in the liver and contributing to the progression of T2D in mice [174]. Remarkably, bEVs can also contain miRNA-like molecules that have been shown to exert trans-kingdom gene expression regulation, in this way influencing host biological functions. For instance, bEV miRNAs of periodontal pathogens can regulate host immune system response by downregulating cytokine expression in Jurkat T cells [175], and *Aggregatibacter actinomycetemcomitans* bEV miRNAs promote human macrophage TNFα production and can even cross the blood-brain barrier in mice, thus evidencing a potential role of bEV miRNAs in neuroinflammatory disorders [176]. Similarly, *Paenalcaligenes hominis* bEVs can also enter the brain and cause cognitive function-impaired disorders [173]. Further studies are needed to understand the systemic functions of circulating bEVs, to explore their potential as biomarkers, and to identify and correlate their taxonomy to the metabolic status of gut microbiota.

#### Properties of bEVs Derived from Probiotics

Probiotics and prebiotics improve the composition of gut microbiota and are postulated as promising tools for the prevention and treatment of obesity [186] (Figure 3). Probiotics consist of live bacteria, typically *Lactobacilli* and *Bifidobacteria*, that promote the health of the host. In particular, probiotics are immuno-modulatory and have beneficial effects such as improved skin health and alleviation of IBD. Moreover, probiotics improve gut permeability and promote fat loss. In fact, obese mice supplemented with *Lactobacillus plantarum* showed a reduction in adipose deposition and enhanced lipid oxidation [187]. Recently, *O. laneus* was proposed as a novel probiotic able to decrease the levels of systemic succinate, which correlate with inflammation and are linked to numerous complications in obese mice [188]. Probiotics also favor microbial diversity, expanding the beneficial bacteria and reducing certain harmful species and their metabolites. In this sense, probiotics increase SCFA-producers and reduce LPS-producing bacteria. Accordingly, HFD rats supplemented with probiotics decreased weight gain and displayed positive effects on insulin and fasting blood glucose, inflammatory markers, and adipokine levels [189].

Notably, probiotic bEVs also possess anti-inflammatory properties and could be potentially used to treat inflammatory disorders, including diabetes [190,191]. For instance, *L. paracasei* bEVs counteract LPS-induced human colorectal cell inflammation and attenuate colitis in mice [181]. Moreover, *L. casei* bEVs participate in the crosstalk between gut microbiota and the modulation of the immune system of the host [182]. Both *L. casei* and *L. plantarum* bEVs’ anti-inflammatory properties can be strengthened after macrophage treatment in vitro [192]. This finding provides a new avenue to analyze if specific food ingredients could enhance probiotic bEV immunomodulatory properties in a similar manner as culture conditions and be used to effectively treat metabolic diseases.

Alternatively, prebiotics are non-metabolized food ingredients that reach the intestinal lumen and can be selectively utilized by beneficial bacteria in the host. Inulin, galacto-oligosaccharides (GOS), and fructo-oligosaccharides (FOS) are the most frequently used prebiotic supplements. FOS, for instance, increases the number of *Bifidobacterium* and *Lactobacillus* species, as well as other SCFA producers, reinforcing the gut barrier against pathogens and leading to improvements in metabolic parameters and a decline in food intake [193]. Leptin resistance is also improved by a single dose of prebiotics, but clinical trials investigating their use in the treatment of obesity are still underway. Interestingly, cocoa flavanols, dark chocolate, and lycopene have a systemic effect on metabolism mediated by the gut microbiota [194]. However, the full potential of prebiotics for weight control and metabolic regulation is still unknown.

### 3.3. Food-Derived EVs as Potential Therapeutic Agents

**Figure 4:**
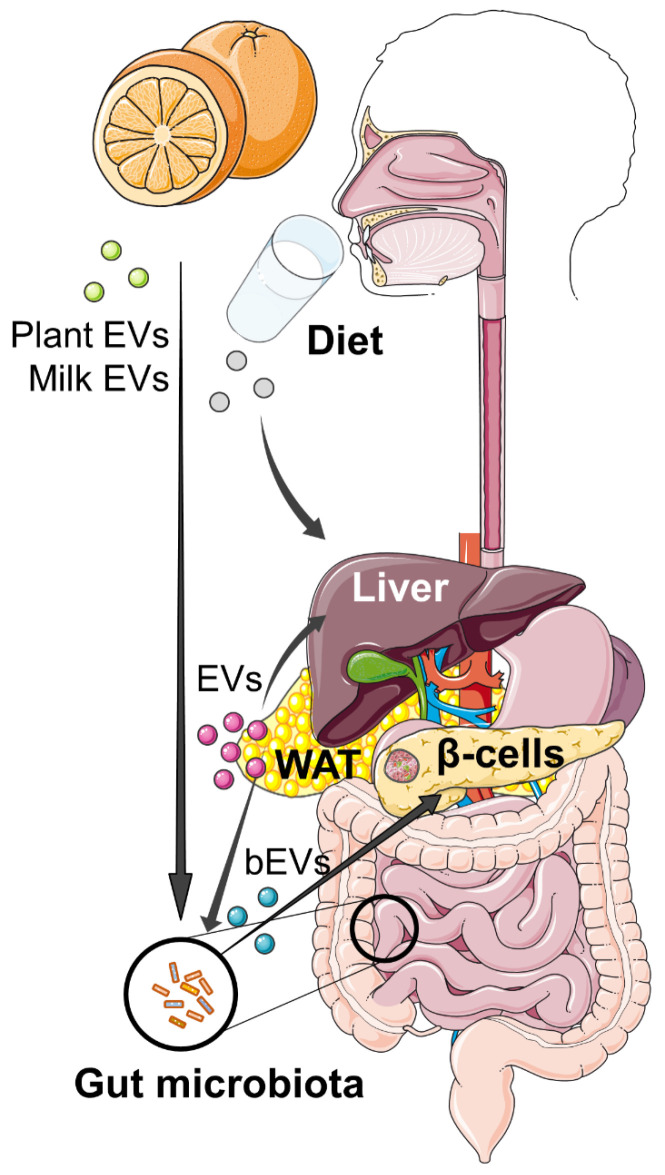
EVs found in edible vegetables or milk, or released by the gut microbiota and the different organs, participate in a multi-directional, inter-kingdom crosstalk that is required to coordinate metabolic processes and maintain homeostasis in the host. See text for details.

As observed for the bEVs derived from probiotics, dietary EVs can participate in the interaction between diet and gut microbiota, thus suggesting that EVs might be involved in the previously described effect of diet on host physiology. Additionally, dietary EVs can directly interact with mammalian cells, such as epithelial cells, immune cells, tumor cells, or hepatocytes, thus mediating biological functions and ultimately impacting on host health (Figure 4) [195,196]. EVs from plants and food are absorbed daily through the intestine. Importantly, EVs have been shown to survive the acidic environment of the digestive tract because of the protection afforded by the phospholipid bilayer [197]. The miRNAs in milk-derived exosomes are still detected after RNase, freeze-thawing, and acidic treatments [198], and curcumin encapsulated in exosomes is four times more stable than free curcumin [199]. The uptake of milk-derived exosomes by intestinal epithelial cells (IECs) is mediated by endocytosis and depends on cell and exosome surface glycoproteins in human and rat intestinal cells [200]. Moreover, EVs from food also cross the endothelial cell barrier of blood vessels and reach distal organs, thus influencing the systemic condition of the host. Oral administration of fluorescently labeled milk exosomes in mice showed accumulation in the liver [201,202].

Recent characterizations of EVs from food show that they might contain pharmacologically active molecules. Therefore, food EVs are attracting increasing interest due to their relevance in modulating cellular processes as well as their potential as therapeutic vehicles for the treatment of a variety of diseases, such as inflammatory disorders, hepatic steatosis, and cancer. For instance, extracellular vesicles isolated from homogenized ginger rhizome roots using a sucrose gradient centrifugation method [203], named ginger-derived nanoparticles (GDNs), were shown to contain large amounts of ginger bioactive constituents (6-gingerol and 6-shogaol). These GDNs were taken up by IECs and macrophages [204]. Oral administration of GDNs naturally containing shogaol protects mice from alcohol-induced liver damage by activating NRF2 [203].

In support of this concept, dietary bovine milk exosomes modulate gut microbiota composition, especially increasing SCFA levels [205]. One of the main consequences of this modulation in mice is the maintenance of intestinal barrier function and the regulation of the intestinal immune system. Actually, maternal breast milk contains abundant exosomes that may function as signalosomes, inducing β-cell proliferation shortly after birth, whereas weaning and stopping the exposure to milk exosomes may represent the key signal for β-cell maturation [206]. However, continued consumption of bovine milk by Western societies, and hence continued exposure to milk exosomes, may have harmful effects on human health since other studies suggest that they also participate in the pathogenesis of metabolic diseases [207]. This is because the most abundant miRNAs in bovine milk exosomes, including *miR-21* and *miR-148a*, disturb energy homeostasis by influencing adipogenesis and insulin secretion, hence adding a new perspective to the pathogenesis of T2D [206]. Alternatively, exosomes from the breast milk of mothers with a metabolic disease may impact infant health. Milk exosomes from T1D mothers are enriched in immune-modulating miRNAs, whose transference to the infant could lead to overactivation of the immune system [208]. Similarly, maternal obesity and gestational diabetes are associated with alterations in the content of specific exosomal miRNAs in human milk that may increase the adiposity of the infant [209,210].

All these data show the potential of using milk exosomes as a scalable, biocompatible, and cost-effective delivery system to enhance the efficacy of therapeutic miRNAs or other drugs that are not orally bioavailable. Milk-derived exosomes loaded with *miR-31* have been tested as a novel system to promote angiogenesis, thus accelerating diabetic wound healing, one of the major health problems worldwide [211]. Most recently, milk EVs carrying the antidiabetic drug Liraglutide have been developed as the next-generation drug delivery platform for the treatment of diabetes [212].

Dietary plant EVs can also modify intestinal communication with distal tissues, including the placenta. Watermelon EVs affect the trophoblast proteome and key aspects of trophoblast behavior, including migration, thus recommending a maternal diet high in fruit and vegetables as it lowers the risk of fetal growth restriction [213]. In addition, dietary plant EVs influence human health by modulating gut microbiota and intestinal barrier function [214]. Moreover, EVs from oranges could be used for the treatment of intestinal complications associated with diet-induced obesity in mice [215]. Similarly, ginger EVs show therapeutic promise. In particular, GDNs block NLRP3 inflammasome assembly and activation, thus emerging as new potent agents to treat the pathogenesis of many diseases, including Alzheimer’s disease and T2D [216]. Recently, GDNs have been shown to regulate the intestinal immune system and improve gut barrier integrity through miRNA delivery in mice [217]. Moreover, manipulation of gut commensal *L. rhamnosus* through dietary GDNs may be a promising strategy to treat metabolic syndrome, and oral administration of GDNs enriched in *miR-375* to HFD mice improves host glucose tolerance and insulin response [218].

There is a growing interest in therapeutically targeting the inflammatory response that underlies age-related chronic diseases, including obesity and T2D, as well as other pathologies, such as autoimmune diseases and neurodegenerative disorders, which have a serious impact on the lives of patients. Interestingly, celery exosomes suppress activated T lymphocytes in a dose-dependent manner, and *miR-159a* and *miR-156c* found in nut exosome-like nanoparticles suppress inflammation. These data suggest that plant EVs can be used as immunosuppressants to treat a variety of ailments [219,220].

## 4. Open Questions and Perspectives

### 4.1. Current Limitations in EV Characterization

All the reports that we have summarized point to a key role for EVs of different origins—plant, bacterial, and mammalian—in the maintenance of metabolic homeostasis and the etiopathology of metabolic diseases. Hence, EVs and bEVs display great potential as biomarkers to diagnose or monitor disease progress, therapeutic targets to alleviate many illnesses, and innovative therapeutic vectors to increase the bioavailability of different drugs [221,222]. However, standard techniques for the isolation of bacterial and eukaryotic vesicles from different types of samples should be well established before EVs reach the clinical setting as either biomarkers or therapeutic agents. Current methodologies for EV isolation do not discriminate between the different types of vesicles, such as MVs or exosomes, as no specific markers exist. Similarly, although some steps have been taken in that direction, there is a need to find strong tissue-specific markers that identify the source of EVs circulating in the blood. Selective isolation of EVs coming from metabolically relevant tissues such as the liver or pancreatic β-cells would provide us with more precise biomarkers than interrogating the whole pool of EVs circulating in blood.

Alternatively, bEVs from gut microbiota are frequently characterized from stool samples. Some authors identify the bacterial origin of fecal bEVs by amplifying DNA from the most common gut microbiota phyla. However, exhaustive characterization is still a challenge due to the complexity of the material. Whereas the characterization of bEVs from culture medium is relatively straightforward, the isolation and characterization of bEVs from the corporal fluids of the host are far more complex. The major obstacle is the lack of bEV universal markers and their similar size to mammalian EVs. Nevertheless, new methods are currently being developed to optimize bEV isolation from body fluids [223]. Additionally, the identification of bioactive molecules in bEVs from gut microbiota is still in its infancy and has been mainly focused on the analysis of the proteome [224]. However, as we have mentioned, bEVs also carry miRNA-like molecules that could potentially regulate gene expression in mammalian cells. Hence, a deeper knowledge of bEVs may provide a novel and unexpected insight into the pathobiology of diabetes.

### 4.2. Perspectives

Aside from optimizing and standardizing methods for EV isolation from different tissues, the gut microbiota, or food ingredients, extensive characterization of their cargo should be performed before using them as therapeutic agents or drug delivery vectors in humans. We and others have used EVs of different origins as carriers of artificial genetic material, miRNAs or siRNAs, for the treatment of obesity and diabetes in rodent models [22,66,225], but as mentioned, EVs from MSCs of different sources have different endogenous properties and induce differential effects on acceptor cells [111]. Hence, exhaustive characterization of EVs with potential therapeutic use would be key to optimizing their effects. Other authors have used food-derived EVs as carriers to deliver bioactive molecules to tissues [212,226], with the advantage that they can be administered orally; however, stronger evidence is still needed to ascertain what portion of EVs from food reach the circulation and directly affect the function of internal organs.

Of the miRNAs mentioned in the text, *miR-122* is the most studied. *miR-122* is a liver-enriched miRNA that participates in the regulation of cholesterol metabolism, and its circulating levels have been shown to positively correlate with liver injury and different features of glucose metabolism and the metabolic syndrome [227]. As mentioned, it also negatively correlates with the Firmicutes/Bacteroidetes ratio. Most of the studies point to a detrimental role of *miR-122* overexpression in metabolism (Table 1), and we and others have shown the beneficial effects of inhibiting it [225,228]. Similarly, *miR-155* is a pro-inflammatory miRNA that has been shown to be released from lymphocytes or macrophages and may participate in the development of insulin resistance in both type 1 and type 2 diabetes [229]. Alternatively, *miR-223-3p* has an anti-inflammatory function and participates in the regulation of lipid and cholesterol homeostasis, delays atherosclerotic plate formation, and decreases steatosis (Table 1) [230,231,232,233]. We recently showed that EV-*miR-223-3p* is significantly decreased in prediabetic subjects that go on to develop T2D in the next 4 years [234].

Biodistribution studies are also required to identify membrane molecules targeting delivery to specific cell types. This information is needed to take full advantage of EVs’ ability to deliver bioactive molecules and drugs, thereby decreasing unwanted secondary effects. Modification of the cells used to produce the EVs by overexpressing specific membrane proteins shows promise in this regard [235].

Additionally, we still need to better understand the mechanisms by which gut microbiota and the cells of the host communicate with each other and collaborate in maintaining energy homeostasis. Thus, more evidence is needed to know if bEVs with bioactive cargo directly affect the function of mammalian cells in the host, aside from inducing inflammation, and conversely, if eukaryotic EVs from the host are able to modulate the composition of the gut microbiota. In this respect, EVs from intestinal epithelial cells have been shown to carry miRNAs that can influence bacterial gene expression, thus controlling gut microbiota composition and homeostasis [236]. However, a deeper knowledge of inter-kingdom crosstalk would show if more distant organs of the host, such as the skeletal muscle or the liver, are also able to communicate with the gut microbiota via EVs. This would give us a mechanism by which lifestyle interventions or environmental factors other than food, such as exercise, fasting, or even depressive moods, may affect gut microbiota composition.

## 5. Conclusions

EVs can be found in all domains of life and act as complex signalosomes that mediate communication between the different tissues and kingdoms to coordinate cellular functions in order to maintain metabolic homeostasis in the host. Alterations of prokaryotic or eukaryotic EVs may lead to metabolic imbalance and disease, whereas their modulation could be used as a novel therapeutic strategy for the alleviation and treatment of metabolic diseases.

## Figures and Tables

**Table 1:** Summary of the studies mentioned in the text dealing with EV-mediated inter-tissue communication. See text for details.

Source of EVs	Relevant Cargo	Target Cell/Tissue	Effect	Ref
ECs	Plasma components	Adipocytes	Signaling	[39]
Adipocytes	SNHG9	ECs	Anti-inflammation	[40]
Adipocytes	*miR-221-3p*	VSMCs	Vascular remodeling	[42]
Adipocytes	*miR-122*	Preadipocytes	Adipogenesis	[43]
Adipocytes	*miR-3604*	Preadipocytes	Reduced adipogenesis	[45]
Adipocytes	*miR-34*, *miR-1224*	Macrophages	M2 polarization inhibition	[46,47]
Adipocytes	ND	Macrophages	Foam cell formation	[48]
Adipocytes	*miR-222*, *miR-27a*	Myocytes	Lower insulin sensitivity	[38,49]
Adipocytes	Mitochondria	Cardiomyocytes	Antioxidant	[50]
Adipocytes	Adipsin	Cardiomyocytes	Attenuated remodeling	[51]
Adipocytes	*let-7d-3p*	Muscle stem cells	Decreased proliferation	[52]
Adipocytes	*miR-199a*, *miR-141-3p*	Hepatocytes	Hepatic steatosis and lower insulin sensitivity	[53,54]
Adipocytes	*miR-9-3p*	Brain	Cognitive impairment	[55]
Brown adipocytes	NPM3	WAT	Browning	[56]
Brown adipocytes	*miR-30b*	Kidney	Reduced fibrosis	[57]
Adipose MSCs	STAT3	Macrophages	M2 polarization	[58]
Adipose MSCs	OPG	Macrophages	Decreased osteoclast differentiation	[59]
Adipose MSCs	*miR-627*, *miR-223-3p*	Hepatocytes	Decreased steatosis	[60]
Adipose MSCs	*miR-21-3p*, *miR-126*	Fibroblasts	Accelerated wound healing	[61,62]
M1 macrophages	*miR-155*, *miR-29a*	Adipocytes, hepatocytes, myocytes	Lower insulin sensitivity	[36,63]
M1 macrophages	*miR-212*, *miR-155*	Pancreatic β-cells	Decreased insulin secretion	[64,65]
M2 macrophages	*miR-690*	Adipocytes, hepatocytes, myocytes	Higher insulin sensitivity	[35]
Muscle	*miR-133b*	Liver	Higher insulin sensitivity	[66]
Muscle	*miR-1*	WAT	Increased lipolysis	[67]
Muscle	*miR-191a*, *miR-27a-3p*	WAT	Browning	[68]
Muscle	*miR-690*	Muscle stem cells	Inhibition of differentiation	[69]
Muscle	*miR-16*	Pancreatic β-cells	Increased proliferation	[70]
Liver	*miR-130a-3p*	Adipocytes	Higher insulin sensitivity	[71]
Liver	TM4SF5	Brown adipocytes	Higher insulin sensitivity	[72]
Liver	miR-3075	Adipocytes, hepatocytes, myocytes	Higher insulin sensitivity	[73]
Liver	*miR-122*	Cardiomyocytes	Impaired mitochondrial function	[74]
Liver	*miR-122*	ECs	Angiogenesis	[75]
Hepatocytes	*miR-122*	Macrophages	Increased inflammation	[76]
Hepatocytes	*let−7b*	Brown adipocytes	Decreased thermogenesis	[77]
Neutrophil	*miR-223-3p*	Hepatocytes	Decreased inflammation and hepatic steatosis	[78]
Pancreatic β-cells	*miR-26a*	Pancreatic β-cells	Improved insulin secretion	[79]
Pancreatic β-cells	IAPP	Hippocampus	Mitochondrial fission	[80]
T lymphocytes	*miR-155*	Pancreatic β-cells	Apoptosis	[81]

**Table 2:** Summary of the studies mentioned in the text dealing with bacteria-host EV-mediated inter-kingdom communication. See text for details.

Source of EVs	Relevant Cargo	Target Cell/Tissue	Effect	Ref
*A. muciniphila*	ND	IECs	Anti-inflammatory	[159]
*A. muciniphila*	ND	IECs	Gut fortification	[170]
*A. muciniphila*	ND	Liver, AT	Anti-obesity	[171,172]
*A. muciniphila*, *EcN1917*	ND	Gut	Colitis protection	[162,163]
*O. splanchnicus*	ND	IECs	Anti-inflammatory	[160]
*B. Fragilis*	PSA	DCs	Colitis protection	[164]
*B. thetaiotaomicron*	anSME	Gut	Colitis	[169]
*P. hominis*	LPS	Hippocampus	Cognitive impairment	[173]
*P. panacis*	LPS	Adipose, muscle	Insulin resistance	[157]
*P. pentosaceus*	ND	Macrophages	Immuno-suppression	[165]
*P. gingivalis*	Proteases	Liver	Impaired glucose metabolism	[174]
*P. gingivalis*, *T. denticola*, *A. actinomycetemcomitans*	miRNAs	T cells	Anti-inflammatory	[175]
*A. actinomycetemcomitans*	miRNA-like	Macrophages	Neuro-inflammatory	[176]
*E. coliC25*	ND	IECs	Pro-inflammatory	[166]
*EcN1917*, *ECOR12*	ND	IECs	Pro-inflammatory	[167]
*EcN1917*, *ECOR63*	ND	IECs	Anti-inflammatory	[161]
*F. prausnitzii*	ND	IECs	Pro-inflammatory	[168]
Gut	mbDNA	Macrophages, Adrenal cells	Hypertension	[177]
Gut	mbDNA	Hepatocytes	Liver fibrosis	[178]
Gut	mbDNA	Liver, adipose, muscle	Pro-inflammatory	[179]
Gut	DNA	β-cell	Insulin resistance	[180]
*L. paracasei*	ND	IECs	Colitis protection	[181]
*L. casei*	ND	IECs	Anti-inflammatory	[182]

## Data Availability

Not applicable.
